# High-Sensitivity Pixels with a Quad-WRGB Color Filter and Spatial Deep-Trench Isolation

**DOI:** 10.3390/s19214653

**Published:** 2019-10-26

**Authors:** Yongnam Kim, Yunkyung Kim

**Affiliations:** Department of Electronics Engineering, Dong-A University, Busan 49315, Korea; kkvvv12@gmail.com

**Keywords:** quad-WRGB, spatial-DTI, CMOS image sensor

## Abstract

The demand for a high-resolution metal-oxide-semiconductor (CMOS) image sensor has increased in recent years, and pixel size has shrunk below 1.0 μm to allow accumulation of numerous pixels in a limited area. However, shrinking the pixel size lowers the sensitivity and increases crosstalk because the aspect ratio is worsened by maintaining the height of the pixel. This work introduces a high-sensitivity pixel with a quad-WRGB (White, Red, Green, Blue) color filter array (CFA), spatial deep-trench isolation (S-DTI), and a spatial tungsten grid (S-WG). The optical performance of the suggested pixel was analyzed by performing 3D optical simulations at 1.0, 0.9, and 0.8 μm pixel pitches as small-sized pixels. The quad-WRGB CFA is compared with the quad-Bayer CFA, and the S-DTI and S-WG are compared with the conventional DTI and WG. We confirmed an improvement in the sensitivity of the suggested pixel using the quad-WRGB CFA with S-DTI and S-WG to a maximum of 58.2%, 67.0%, and 66.3% for 1.0, 0.9, and 0.8 μm pixels, respectively.

## 1. Introduction

The advances in camera technology now necessitate the development of a high-resolution complementary metal-oxide-semiconductor (CMOS) image sensor. However, a high-resolution camera requires a pixel pitch smaller than 1.0 μm to allow accumulation of numerous pixels in the limited sensor area [1]. Accordingly, the aspect ratio is worsened by maintaining the pixel height in a small pixel pitch. If the pixel depth gets thinner, the color filter (CF) or the photodiode should also be thinner. However, the thickness of the CF has pigment limitations [2], and making the photodiode thinner is difficult because of the lower light absorption at long wavelengths [3]. Therefore, the optical performance of a small-sized pixel deteriorates due to low sensitivity and high crosstalk, which lead to a lower performance of CMOS image sensors.

Many approaches are available for enhancing the sensitivity and crosstalk for a small pixel. Particularly good examples include new optical pixel structures, such as the stacked grid structure between color filters, the lensed CF, and the front-inner lens image sensor [4,5,6]. The stacked grid structure is located between the color filters, and it blocks the optical crosstalk to increase sensitivity [4]. However, the grids cannot be adapted to small pixels because the CF width reduces and adversely affects the sensitivity by shrinking the absorption area. In [5], a lensed CF is proposed to reduce crosstalk and enhance the sensitivity by uniting the micro lens and a color filter. However, the height and the radius of curvature (ROC) of this structure are difficult to process equally. The front-inner lens is proposed to enhance the sensitivity by adding a lens to the front side to serve as the interconnection layer in a shallow photodiode [6]. However, the complicated structure of the front inner lens creates substantial difficulty in conducting general processes.

Conversely, different approaches are available to obtain high sensitivity by changing the color filter array (CFA) [7,8,9,10]. A CFA with a white color filter has been studied because a white color filter transmits incident light at all wavelengths. The WRGB (white, red, green, blue) CFA indicates that one of the green pixels of the Bayer CFA was replaced with a white pixel [7]. The RGBW CFA consisting of 50% white CF has also been developed to improve the sensitivity and resolution [8]. Fifty percent of the total area of the RGBW CFA is occupied by the white CF, 25% by the green CF, and 12.5% by the red and blue CF. The RGBW CFA consisting of 75% white CF with a color separation process was also introduced [9,10]. The low-illumination signal-to-noise ratios of the luminance signal are improved by increasing the white CF area [11]. Moreover, the color reproducibility of the WRGB pattern is comparable with the Bayer pattern [11,12]. According to this research, the WRGB CFA demosaicing result has a similar spatial resolution, color reproduction, and better color aliasing artifact levels compared to the Bayer CFA demosaicing result by the post-processing method. However, much crosstalk is induced with increasing sensitivity, especially for a small pixel. Because the white color filter transmits a lot of light, some of the light enters the other pixel’s photodiode. This problem still requires a solution.

In this work, we propose a quad-WRGB structure with spatial-deep trench isolation (S-DTI) and a spatial-tungsten grid (S-WG) to solve the small pixel problems, such as low sensitivity and high crosstalk. The quad-WRGB CFA provides a four-fold expansion of each color filter. The S-DTI and S-WG are located every four pixels for efficient reduction of the crosstalk. The optical performance is investigated by simulating the suggested structure and comparing it with typical structures with the CFA, DTI, and WG using a commercial 3D optical simulator for precise analysis. Section 2 describes the concept of the quad-RGBW pixel structure having S-DTI and S-WG, and Section 3 presents the simulation results and discusses the results. Conclusions are presented in Section 4.

## 2. Concepts of the Quad-WRGB Color Filter Array and Spatial DTI

**Figure 1:**
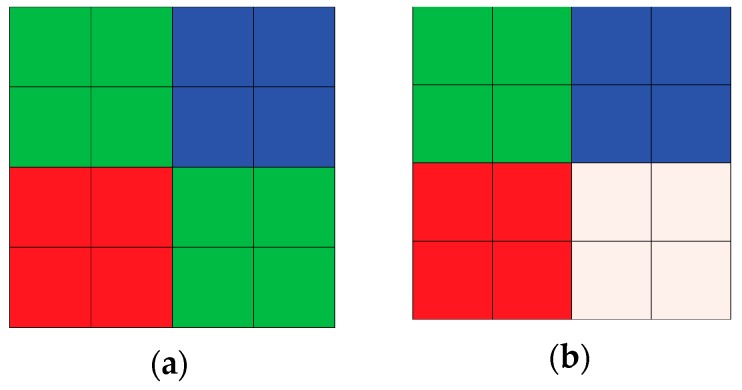
Color filter arrays in 4 × 4 pixels: (**a**) Quad-Bayer array; (**b**) Quad-WRGB array. The same color filter is arranged in 2 × 2 pixels.

As previously mentioned, the aspect ratio worsens with shrinking pixel size in high-resolution camera. Accordingly, the crosstalk increases due to the narrower and longer optical path and the sensitivity decreases because of the narrowed pixel pitch. Therefore, the sensitivity in low light environments becomes increasingly worse because of poor light incidence. The quad-Bayer filter is used for high sensitivity, especially in low light, even though the resolution is effectively divided by four [13,14]. The signals from the four adjacent pixels with the same-colored filter are added together, thereby enabling a brighter image with low noise. In this paper, we introduce a quad-WRGB array that gives a higher sensitivity than the quad-Bayer array. Figure 1 shows the quad-Bayer and the quad-WRGB array; in the latter, the 25% green filter is changed to a white filter. The sensitivity is enhanced because the white color filter has high transmittance at all wavelengths.

**Figure 2:**
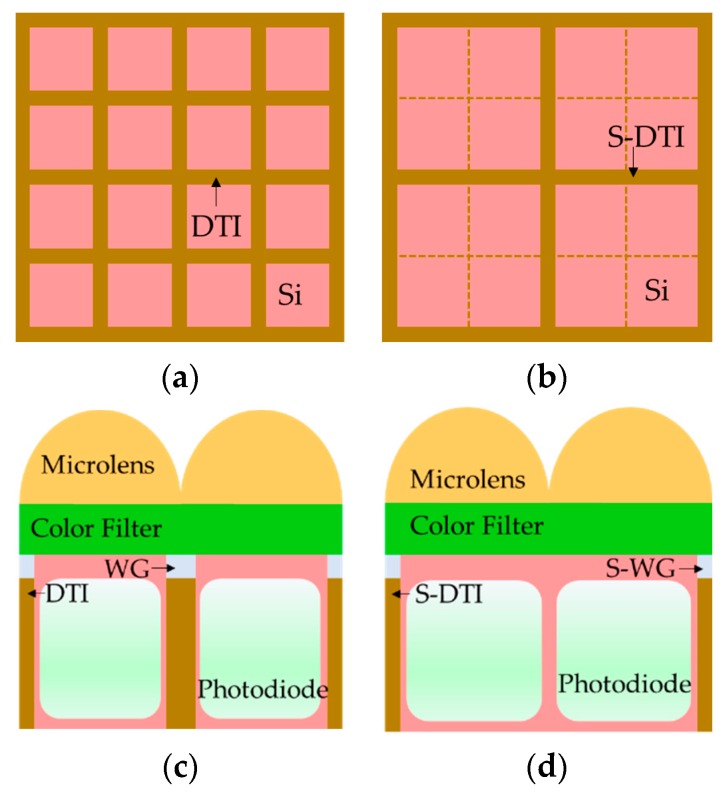
Pixel structures with deep trench isolation (DTI) and tungsten grid (WG): (**a**) Top view of the silicon with DTI. The DTI has located each pixel; (**b**) Top view of the silicon with spatial deep-trench isolation (S-DTI). The S-DTI has located four pixels; (**c**) 2D cross-section of pixels with DTI and WG; (**d**) 2D cross-section of pixels with S-DTI and S-WG.

Conversely, deep trench isolation (DTI) and tungsten grid (WG) are commonly used to block crosstalk [15]. DTI usually blocks crosstalk in the silicon layer, and WG blocks the crosstalk in the color filter planarization layer. DTI and WG, as shown in Figure 2a,c, are located between the pixel and the adjacent pixel. In this work, we propose a spatial DTI and spatial WG for an adaptable quad-WRGB array. As shown in Figure 2b,d, the proposed S-DTI and S-WG block the boundary facing the other color pixels except those facing the same color pixel. In Figure 2, the pink area indicates the silicon area, and the white area is the photodiode. There is no physical isolation without DTI and WG between adjoined same color pixels in Figure 2b. S-DTI refers to the DTI surrounding the four same-colored pixels, except between those pixels, as shown in Figure 2b. Likewise, S-WG refers to the tungsten grid (WG) surrounding the four same-colored pixels, except between those pixels, as shown in Figure 2d. The S-DTI and S-WG help to ensure that the crosstalk is passed through between the same color pixels and that the undesired crosstalk is blocked between the different color pixels. Because the DTI and WG block every instance of crosstalk entering the pixel, the DTI and WG match more closely with the typical Bayer CFA than with the quad-Bayer CFA. In addition, unlike the DTI and WG, the available area for light absorption in the pixel is expanded, because the S-DTI and S-WG occupy the outside part of the four pixels, as shown in Figure 2c,d. Thus, the expanded available area of the S-DTI, S-WG structure increases the light absorption better than the DTI, WG structure. Therefore, the S-DTI and S-WG structure with the quad-WRGB CFA can promise an improvement in the pixel sensitivity by increasing the available area.

## 3. Simulation Results

**Figure 3:**
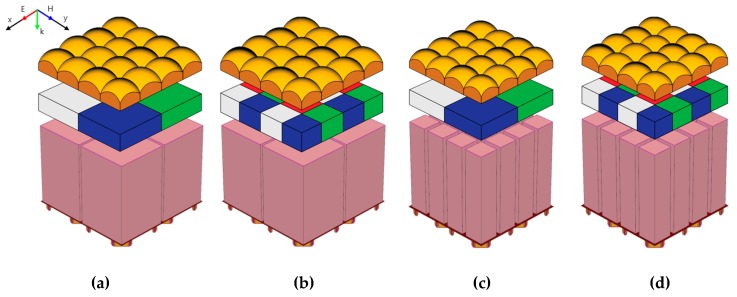
The simulated 3D Pixel structures. 4 × 4 pixel arrays. The microlens, CFA, and photodiodes are separated for ease of viewing. (**a**) Quad-Bayer CFA with DTI and WG, (**b**) Quad-Bayer CFA with S-DTI and S-WG, (**c**) Quad-WRGB CFA with DTI and WG, and (**d**) Quad-WRGB CFA with S-DTI and S-WG.

Numerical analysis of the suggested structure was conducted using the finite-difference time-domain (FDTD) method [16]. This method is broadly used for the optical analysis of CMOS image sensors [17]. The pixel pitches were 1.0, 0.9, and 0.8 μm for the small pixel size. The depth of the silicon as a photodiode was 3.0 μm and the thickness of the color filter was 0.6 μm. The depths of the DTI and WG were 3.0 and 0.2 μm, respectively. The depth of DTI and WG are the same thickness as the silicon and color filter planarization layer for the full-isolation. The width of the WG was 50 nm. The height of the microlens was optimized for high sensitivity at 0.6 μm for the 1.0 and 0.9 μm pixels and 0.4 μm for the 0.8 μm pixel. Likewise, the radius of curvature (ROC) of the micro lens was optimized at 0.8 μm for the 1.0 and 0.9 μm pixels and 0.5 μm for the 0.8 μm pixel. The DTI and WG widths were also optimized for high sensitivity, as shown in Table 1. The DTI and WG were simulated with the varied width from 50 nm to 125 nm. Then, the width of them is determined by the highest photon density. The simulated conditions of each pixel pitch and pixel type are shown in Table 1. The wavelengths of the incident light were 650, 540, and 450 nm as red, green, and blue light, respectively. The sensitivity in oblique incident light was investigated by setting the incident angles to 0°, 10°, and 20°. In addition, the light entered horizontally and vertically, as shown by the *x*- and *y*-axis in Figure 3. Figure 3 shows the 3D simulated structures. The proposed structure shown in Figure 3d is compared with Figure 3a–c. The quad-WRGB CFA is compared with the quad-Bayer CFA, and the S-DTI and S-WG are compared with the DTI and WG. Therefore, four types of pixel structures are simulated, as shown in Figure 3. We first evaluated the optical performance of the quad-Bayer and WRGB CFA with the DTI and WG. We could then confirm which CFA pattern gave the best improvement of the optical performance of the pixel. We then compared the performance between DTI, WG and S-DTI, S-WG.

**Figure 4:**
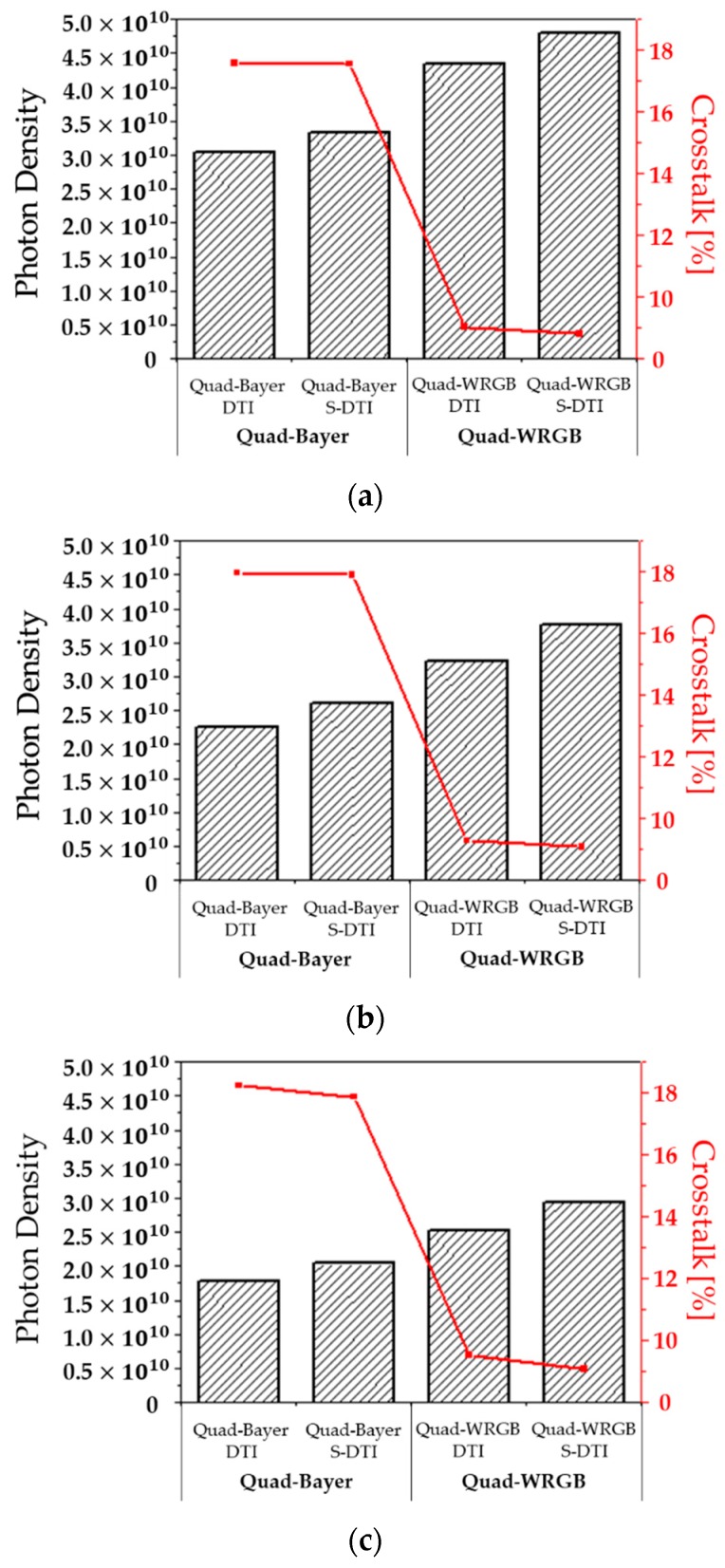
Simulation results of the photon density and the crosstalk in the 1.0 μm, 0.9 μm, and 0.8 μm pixel pitches. The photon density is shown by the black diagonal striped bars, and the crosstalk is shown by the red solid lines. (**a**) 1.0 μm pixel, (**b**) 0.9 μm pixel, (**c**) 0.8 μm pixel.

Figure 4 shows the photon density and the crosstalk for the simulation results of the four types of pixels having the quad-Bayer CFA, quad-WRGB CFA with DTI and WG, or S-DTI and S-WG for the 1.0, 0.9, and 0.8 μm pixel pitches. The photon density represents the light absorbed from the pixels, as shown by the bars. The photon density is the result of adding the results for the wavelength of 650, 540, and 450 nm. These results show that the photon density is improved in all cases by changing the CFA from the quad-Bayer to the quad-WRGB. For all pixel pitches, about 43% of the photon density is enhanced between the quad-Bayer CFA and quad-WRGB CFA with DTI and WG by the white color filter. S-DTI and S-WG also affect the enhancement of photon density, as the enhancement increases as the pixel pitches decrease in size. The maximum improvement of the photon density by S-DTI and S-WG was 16.8% in the quad-WRGB CFA for the 0.8 μm pixel. The minimum improvement of the photon density was 9.7% in the quad-Bayer CFA for the 1.0 μm pixel. The changes in photon density with improved sensitivity of the pixel were a maximum of 58.2%, 67.0%, and 66.3% for the 1.0, 0.9, and 0.8 μm pixels with the quad-WRGB CFA, S-DTI, and S-WG. The crosstalk, which is absorbed light from the next pixel, is represented by the lines in Figure 4. The crosstalk is the rate between the unwanted photon density and the total photon density. As shown in Figure 4, the crosstalk is reduced by S-DTI and S-WG for all types of pixels. The crosstalk was reduced by S-DTI and S-WG at 0.02%, 0.08%, and 0.37% for the 1.0, 0.9, and 0.8 μm pixels, respectively, with the quad-Bayer CFA. The crosstalk was also reduced by S-DTI and S-WG by 0.21%, 0.22%, and 0.45% for the 1.0, 0.9, and 0.8 μm pixels, respectively, with the quad-WRGB CFA. In this work, the crosstalk in the same color filter can be used a good crosstalk as the absorbed photon density. Therefore, we see that the good crosstalk can be absorbed as a signal. This concept is based on the pixel binning which is a process to combine the readout signals of the four same color pixels at a time in the quad-Bayer CFA [13,14]. Therefore, the crosstalk is reduced and the sensitivity is improved. For this pixel binning, the S-DTI/S-WG is useful by passing the crosstalk to the next same color pixel. In addition, the reason that the quad-WRGB CFA has the lower crosstalk than the quad-Bayer CFA is the white pixel which is regarded as having no crosstalk. This is because the full wavelength of the incident light can be absorbed in the white color pixel.

**Figure 5:**
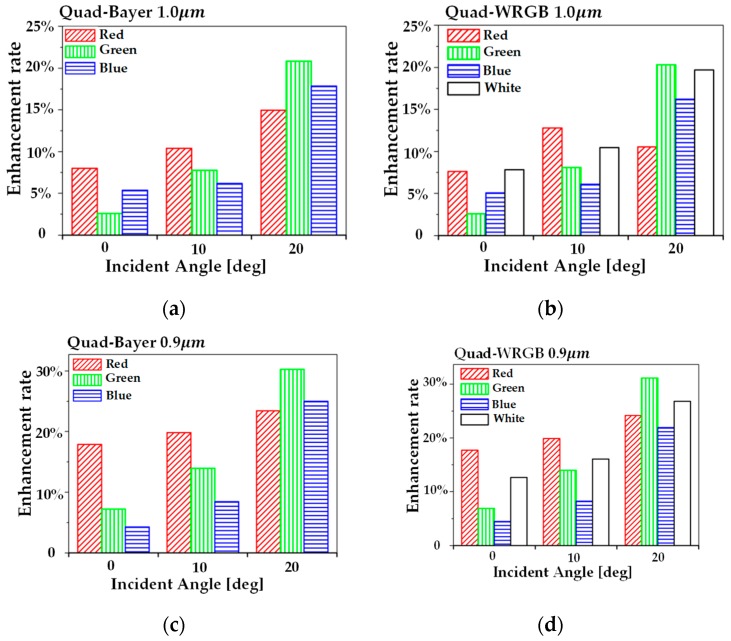

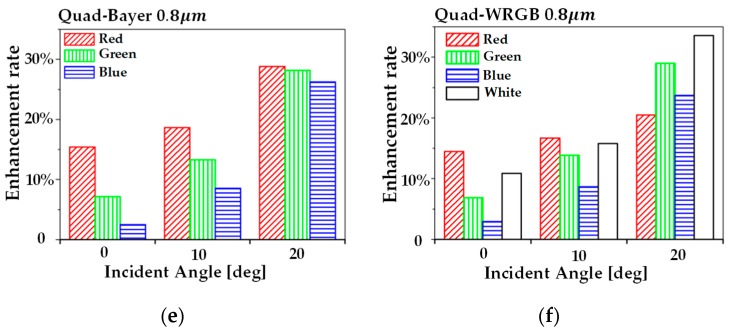
Enhancement rates of the photon density between the DTI/WG and the S-DTI/S-WG at incident angles of 0°, 10°, and 20°. The red oblique striped bar, the green vertical striped bar, the blue horizontal striped bar, and the blank bar indicate the enhancement rates in the red, green, blue, and white pixels, respectively: (**a**) quad-Bayer CFA for the 1.0 μm pixel, (**b**) quad-WRGB for the 1.0 μm pixel, (**c**) quad-Bayer CFA for the 0.9 μm pixel, (**d**) quad-WRGB for the 0.9 μm pixel, (**e**) quad-Bayer CFA for the 0.8 μm pixel, (**f**) quad-WRGB for the 0.8 μm pixel.

A comparison of the sensitivity improvement by the incident angle and pixels is shown by the enhancement rate of the photon density depicted in Figure 5. The enhancement rate is obtained from the ratio of the photon density between the pixel with DTI/WG and the pixel with S-DTI/S-WG. The red oblique striped bar, the green vertical striped bar, the blue horizontal striped bar, and the blank bar indicate the enhancement rates in the red, green, blue and white pixels, respectively. Figure 5a,c,e show the results for pixels having the quad-Bayer CFA, and Figure 5b,d,f show the results for pixels with the quad-WRGB CFA. Figure 5a–f indicate the 1.0, 0.9, and 0.8 μm pixel pitches, respectively. The photon density as the sensitivity of the pixel is enhanced to a maximum of 20.8% for the quad-Bayer CFA and 20.3% for the quad-WRGB CFA in 1.0 μm pixel. The best enhancement rates for the 0.9 μm pixel are 30.3% of quad-Bayer CFA and 31.2% of quad-WRGB CFA. The best enhancement rates for 0.8 μm pixel are 28.8% of quad-Bayer CFA and 33.6% of quad-WRGB CFA. These results, shown in Figure 5, confirm that every photon density is improved by S-DTI and S-WG for every incident angle. The photon density is enhanced more in the oblique incident light. This is because crosstalk, which gets worse in oblique incident light, is used efficiently as the photon density in the same color pixel.

**Figure 6:**
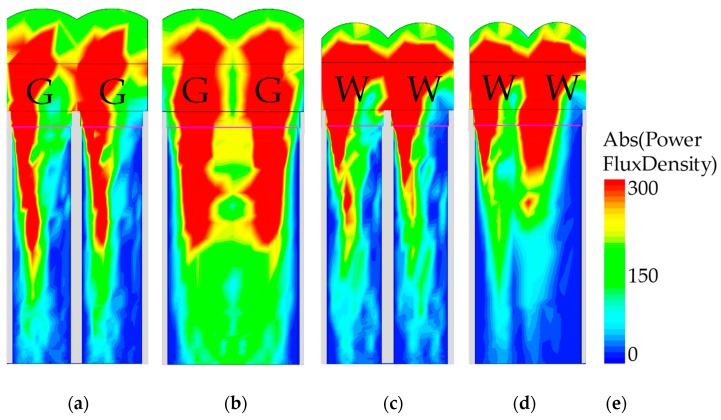
The beam profiles of the simulated power flux density. The incident light was a 540 nm wavelength at 20°: (**a**) the green pixels of the quad-Bayer CFA with DTI/WG in a 0.9 μm pixel, (**b**) the green pixels of the quad-Bayer CFA with S-DTI/S-WG in a 0.9 μm pixel, (**c**) the white pixels of the quad-WRGB CFA with DTI/WG in a 0.8 μm pixel, (**d**) the white pixels of the quad-WRGB CFA with S-DTI/S-WG in a 0.8 μm pixel, (**e**) the scale bar of power flux density. The red region in the beam profile shows the most transmitted light, which could be absorbed in the photodiode.

Figure 6 shows the beam profile of 0.9 and 0.8 μm pixels when a 540 nm wavelength was incident at 20°. The beam profile indicates the power flux density of the transmitted light distribution. Simply stated, the red region in the beam profile shows the most transmitted light, which could then be absorbed by the photodiode. Figure 6a,b show the cross-sections of green pixels with quad-Bayer CFA for the 0.9 μm pixel. Figure 6a,b describe the pixels with DTI/WG and S-DTI/S-WG, respectively. The DTI and WG are shown by the grey color in the beam profile. Figure 6c,d show the cross-sections of white pixels with quad-WRGB CFA for the 0.8 μm pixel. Figure 6c,d describe the pixels with DTI/WG and S-DTI/S-WG, respectively. The beam merged in the pixels with the S-DTI/S-WG, because the S-DTI/S-WG does not locate the DTI/WG in the middle of two same-colored pixels. As mentioned in Section 3, the crosstalk from adjoined same color pixels is absorbed as a signal. The absorption of the crosstalk is shown in Figure 6b,d which has the S-DTI/S-WG. In addition, the crosstalk from the other color pixel is blocked. Therefore, the crosstalk can be use efficiently in the quad-Bayer and quad-WRGB CFA structures. The crosstalk causes the photon density to be absorbed as a signal, thereby improving the pixel performance.

**Figure 7:**
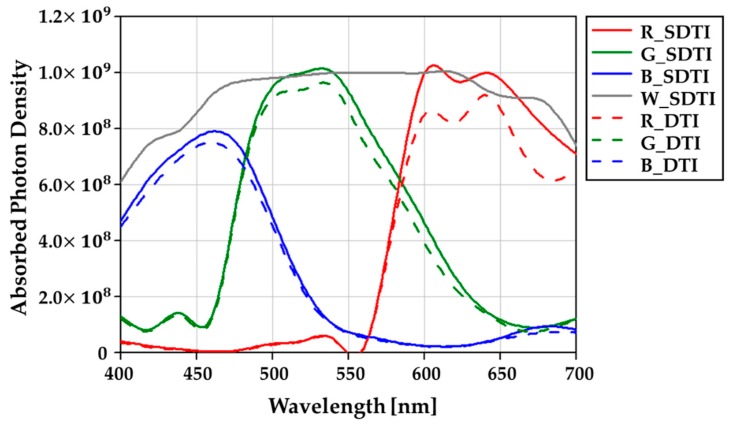
The simulated absorbed photon density with the wavelengths from 400 nm to 700 nm. The light is incident at 0°. The solid line shows the results of the quad-WRGB CFA with S-DTI/S-WG. The dotted line shows the results of the quad-Bayer CFA with DTI/WG. The pixel size is a 0.8 μm.

For clearly showing the enhancement, the absorbed photon density by the wavelength is shown in Figure 7. The absorbed photon density of the quad-WRGB CFA with S-DTI/S-WG and the quad-Bayer CFA with DTI/WG are compared in the 0.8 μm pixel. The light is incident at 0° with the wavelengths from 400 nm to 700 nm. The absorbed photon density of green pixels in the quad-Bayer CFA is averaged. As shown in Figure 7, the absorbed photon density is enhanced in all pixels of the quad-WRGB CFA with S-DTI/S-WG. The maximum improvement is 28% of 680 nm wavelength in a red pixel. We confirm that the quad-WRGB CFA and the S-DTI/S-WG improve the sensitivity efficiently.

## 4. Conclusions

In summary, a high sensitivity and low crosstalk pixel structure having quad-WRGB CFA with S-DTI and S-WG has been introduced. The quad-WRGB CFA indicates a four-fold expansion of the WRGB CFA. The sensitivity is improved by binning the photon from the four pixels having the same CF and by using white CF. The use of spatial DTI (S-DTI) and spatial WG (S-WG) surrounding the four same-colored pixels is suggested for efficient blocking of the crosstalk. The S-DTI and S-WG can block the signals only from different CF; therefore, the absorption area increases for the same CF pixels. The performance of the suggested structure was investigated by 3D optical simulation using 16 × 16 backside-illuminated pixels having the quad-Bayer CFA and the quad-WRGB CFA structure with DTI/WG and S-DTI/S-WG for the 1.0, 0.9, and 0.8 μm pixel pitches. The simulation results indicated that the sensitivity, which is the photon density absorbed in the photodiode, is improved by over 42% by changing from the quad-Bayer CFA to the quad-WRGB CFA for all pixel sizes. Moreover, the sensitivity is improved by 58.2%, 67.0%, and 66.3% by adding S-DTI and S-WG to the quad-WRGB CFA pixels of 1.0, 0.9, and 0.8 μm pixel pitches. The crosstalk is also enhanced by 0.21%, 0.22%, and 0.45%, respectively, by changing the DTI/WG to S-DTI/S-WG for the 1.0, 0.9, and 0.8 μm pixels with the quad-WRGB CFA. Therefore, the quad-WRGB CFA pixel structure with S-DTI and S-WG can promise high sensitivity for small pixels.

The advances in camera technology require the development of a high-resolution camera. Thus, a high-resolution CMOS image sensor is developed in a small pixel pitch as below 1.0 μm to allow accumulation of numerous pixels in the limited sensor area. However, the aspect ratio is worsened in a small pixel pitch and causes low sensitivity and high crosstalk. Although the various CFAs are developed, some CFAs such as quad-Bayer were not preferred, caused by deterioration of resolution. According to advance of compensating resolution technology such as pixel binning, novel demosaicking method, CFAs consisting of the adjacent same color filter are gradually preferred. However, a high crosstalk is still a problem despite solving low sensitivity. Therefore, the optical pixel structure having high sensitivity needs to be developed in a very small pixel without any deterioration. We suggested the novel DTI and WG for the quad-CFA and a small pixel. The suggested S-DTI and S-WG not only induce to absorb the crosstalk from the same color pixels, but also block the crosstalk causing color error from the different color pixels. Moreover, the available area for light absorption in the pixel is expanded, because the S-DTI and S-WG occupy the outside part of the four pixels. Therefore, our work is meaningful for the very small pixels, such as next generation image sensor, as well as 0.7, 0.8, and 0.9 μm pixels.

## Figures and Tables

**Table 1:** Simulation conditions.

Type	Pixel Pitch	Height of Microlens	ROC * of Microlens	DTI * Width
Quad-Bayer CFA with DTI and WG *	1 μm, 0.9 μm, 0.8 μm	0.6 μm (1.0, 0.9 μm pixels) 0.4 μm (0.8 μm pixel)	0.8 μm (1.0, 0.9 μm pixels) 0.5 μm (0.8 μm pixel)	75 nm (all pixel pitches)
Quad-Bayer CFA with S-DTI and S-WG				50 nm (1.0 μm pixel) 75 nm (0.9, 0.8 μm pixels)
Quad-WRGB CFA with DTI and WG				75 nm (all pixel pitches)
Quad-WRGB CFA with S-DTI and S-WG				50 nm (1.0 μm pixel) 75 nm (0.9, 0.8 μm pixels)

* ROC: radius of curvature, * WG: tungsten grid, * DTI: deep-trench isolation.
